# Chimeric immune checkpoint protein vaccines inhibit the tumorigenesis and growth of rat cholangiocarcinoma

**DOI:** 10.3389/fimmu.2022.982196

**Published:** 2022-10-20

**Authors:** Yi-Ru Pan, Chiao-En Wu, Wen-Kuan Huang, Ming-Huang Chen, Keng-Hsueh Lan, Chun-Nan Yeh

**Affiliations:** ^1^ Department of Surgery and Liver Research Center, Chang Gung Memorial Hospital, Chang Gung University, Taoyuan, Taiwan; ^2^ Division of Hematology-Oncology, Department of Internal Medicine, Chang Gung Memorial Hospital, Linkou, Chang Gung University College of Medicine, Taoyuan, Taiwan; ^3^ Center for Immuno-Oncology, Department of Oncology, Taipei Veterans General Hospital, Taipei, Taiwan; ^4^ School of Medicine, National Yang Ming Chiao Tung University, Taipei, Taiwan; ^5^ Division of Radiation Oncology, Department of Oncology, National Taiwan University Hospital, Taipei, Taiwan

**Keywords:** cholangiocarcinoma, immune checkpoint proteins, protein vaccine, immune modulation, therapeutic vaccine, preventive vaccine

## Abstract

Cholangiocarcinoma (CCA) is the second most common primary liver malignancy and carries a dismal prognosis due to difficulties in achieving an optimal resection, and poor response to current standard-of-care systemic therapies. We previously devised a CTLA4-PD-L1 DNA cancer vaccine (DNA vaccine) and demonstrated its therapeutic effects on reducing tumor growth in a thioacetamide (TAA)-induced rat intrahepatic CCA (iCCA) model. Here, we developed a CTLA4-PD-L1 chimeric protein vaccine (Protein vaccine), and examined its effects in the rat iCCA model. In a therapeutic setting, iCCA-bearing rats received either DNA plus Protein vaccines or Protein vaccine alone, resulting in increased PD-L1 and CTLA-4 antibody titers, and reduced iCCA tumor burden as verified by animal positron emission tomography (PET) scans. Treating iCCA-bearing rats with Protein vaccine alone led to the increase of CTAL4 antibody titers that correlated with the decrease of tumor SUV ratio, indicating regressed tumor burden, along with increased *CD8* and granzyme A (*GZMA*) expression, and decreased PD-L1 expression on tumor cells. In a preventive setting, DNA or Protein vaccines were injected in rats before the induction of iCCA by TAA. Protein vaccines induced a more sustained PD-L1 and CTLA-4 antibody titers compared with DNA vaccines, and was more potent in preventing iCCA tumorigenesis. Correspondingly, Protein vaccines, but not DNA vaccines, downregulated PD-L1 gene expression and hindered the carcinogenesis of iCCA. Taken together, the CTLA4-PD-L1 chimeric protein vaccine may function both as a therapeutic cancer vaccine and as a preventive cancer vaccine in the TAA-induced iCCA rat model.

## Introduction

Cancer immunotherapy activates the patient's immune system to attack cancers. The blockade of immune checkpoints is the principal approach of cancer immunotherapy (1, 2). Immune checkpoint proteins such as cytotoxic T lymphocyte antigen 4 (CTLA-4) and programmed death 1 (PD-1)/PD-1 ligand (PD-L1) axis modulate immune responses by negatively regulating T cells (3, 4); however, this mechanism may impair anti-tumor immune responses in patients with malignancy. On top of the success of anti-CTLA-4 antibody, ipilimumab, and antibodies against PD-1 and PD-L1, trials combining both ipilimumab and nivolumab demonstrated encouraging results in advanced melanoma (2, 5). Combining these two antibodies showed a manageable safety profile without additive toxicity (6, 7).

CCA is the second most common liver cancer with aggressive biological behavior and is typically diagnosed at an advanced stage with a poor prognosis (8). According to the original sites, CCAs are divided into three subtypes: intrahepatic CCA (iCCA), perihilar CCA (pCCA), and distal CCA (dCCA) (8). Gemcitabine (GEM)-based chemotherapy is the standard of care for CCA (9–11), but primary or acquired resistance to GEM compromised the therapeutic efficacy (12–14). GEM-based chemotherapy for CCA can achieve an overall response rate of 20-30% (15). After the failure of first-line gemcitabine and cisplatin chemotherapy, there is no standard of care for advanced CCA so far. Cancer immunotherapy, using the patient's immune system to attack cancer, is becoming an increasingly active therapeutic approach. Previous studies showed that infiltration of immunosuppressive immune cells is associated with poor prognosis in CCA patients (16). Pembrolizumab, an anti-PD-1 antibody, is the only FDA-approved immunotherapy. However, in the clinical trials (NCT02628067 and NCT02054806), pembrolizumab monotherapy provided durable response rates in 6–13% of advanced BTC patients (17). In light of these encouraging results, clinical trials are ongoing testing the combination of immune checkpoint inhibitors (ICIs) with GEM-based chemotherapy (18). For example, TOPAZ-1 study recently showed a positive result that durvalumab plus chemotherapy demonstrated longer survivals than those achieved with traditional chemotherapy (19). Thus, it is crucial to explore and establish a suitable and effective anticancer treatment for advanced CCA patients. In this study, the TAA-induced rat iCCA model has been used since 2004 after the first publication in our group (20). This model mimics human iCAA to be used as an animal model for studying iCCA. This model has been repeatedly validated to serve as a powerful pre-clinical platform for therapeutic and chemoprevention strategies for human iCCA.

Cancer vaccine strategies have been in clinical practice or yielded positive results, e.g., DNA vaccines such as HPV vaccines for cervical cancer and HBV vaccines for hepatocellular carcinoma, and peptide/protein vaccines such as HER2 for breast cancer and MUC1 for non-small cell lung cancer and prostate cancer (21–25). CTLA-4 and PD-L1 blockers have been investigated in several trials of melanoma, non-small-cell lung cancer, breast cancer, colorectal cancer, etc. (26–29). Therefore, these immune checkpoint proteins are promising targets for the development of cancer vaccines.

In our previous publication (30), therapeutic CTLA4-PD-L1 DNA cancer vaccines can reduce iCCA tumor growth in a rat thioacetamide (TAA)-induced iCCA model. Vaccine therapy is a cost-effective approach to provide anticancer immunity *via* the stimulation of the immunity against both CTLA-4 and PD-1/PD-L1. Therefore, we further investigated whether CTLA4-PD-L1 DNA or proteins act as the therapeutic and preventive cancer vaccines in a rat iCCA model.

## Materials and methods

### The constructions of CTLA4-PD-L1DNA and protein vaccines

The CTLA4-PD-L1-I DNA vaccine was generated by inserting the IL2 DNA sequence (ATGTATAGGATGCAACTGCTGTCTTGCATTGCTCTGTCTCTGGCACTGGTCACTAACTCTGCC), *ctla4* nt 106-483, and *cd274* nt 55-381 into the pVAC1 vector (**Figure S1A**). The CTLA4-PD-L1-II DNA vaccine was generated by inserting the IL2 DNA sequence, *ctla4* nt 106-483, and *cd274* nt 55-711 into the pVAX1 vector (**Figure S1B**). The purity of pVAC1-IL2-CTLA4-PD-L1-I or pVAX1-IL2-CTLA4-PD-L1-II was determined by the OD260/OD280 ratio, and agarose gel electrophoresis and the accuracy of DNA sequences were determined by DNA sequencing. For CTLA4-PD-L1-I protein vaccine, the IL2 DNA sequence, *ctla4* nt 106-483, and *cd274* nt 55-381 were inserted into pET56 vector (**Figure S1A**). For CTLA4-PD-L1-II protein vaccine, the IL2 DNA sequence, *ctla4* nt 106-483, and *cd274* nt 55-711 were inserted into pET56 vector (**Figure S1B**). His-tagged CTLA-4-PD-L1 protein vaccines were purified by Ni-NTA (Nickel Nitrilotriacetic acid) beads.

### Thioacetamide (TAA)-induced iCCA rat model

**Figure 1:**
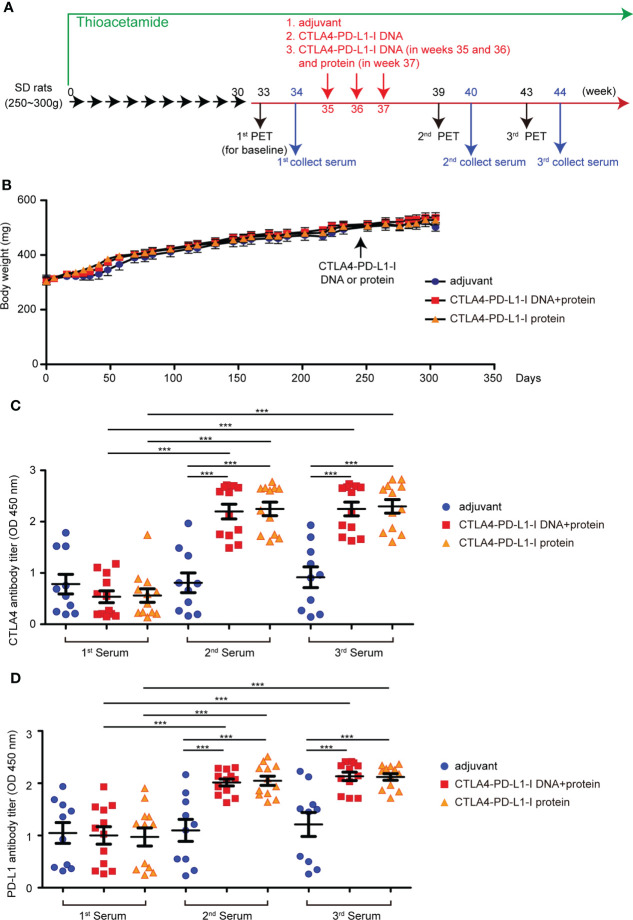
The effect of CTLA4-PD-L1-I DNA or protein vaccine. **(A)** A schema for experimental design for **Figure 1**. **(B)** The average body weight of rats (N≥10) receiving TAA in each experiment arm for 320 days. **(C)** The CTLA4 antibody titers expressed as the OD450 nm were determined after six weeks (2^nd^) and ten weeks (3^rd^) of CTLA4-PD-L1-I DNA, CTLA4-PD-L1-I protein, or control adjuvant (Freund’s adjuvant). N≧10. The values are from two independent experiments and presented as the mean ± SEM. ***P< 0.001 by Student’s *t*-tests. **(D)** The PD-L1 antibody titers expressed as the OD450 nm were determined after six weeks (2^nd^) and ten weeks (3^rd^) of CTLA4-PD-L1-I DNA, CTLA4-PD-L1-I protein, or control adjuvant (Freund’s adjuvant). N≧10. The values are from two independent experiments and presented as the mean ± SEM. ***P< 0.001 by Student’s *t*-tests.

The protocol of animal experiments in this study has been reviewed and approved by the Institutional Animal Care and Use Committee of Chang Gung Memorial Hospital (Approval No: 2015121001 and 2019092403), and recognized that the proposed animal experiment follows the Animal Protection Law by the Council of Agriculture, Executive Yuan, R.O.C. and the guideline as shown in the Guide for the Care and Use of Laboratory Animals as promulgated by the Institute of Laboratory Animal Resources, National Research Council, U.S.A. Thioacetamide (TAA)-induced rat cholangiocarcinoma model is described and validated in our previous papers (30–32). For **Figure 1**, male Sprague-Dawley (SD) rats were fed with drinking water with TAA 300 mg/L for 30 weeks before the vaccinations. For **Figure 3**, male SD rats were fed with drinking water with TAA 300 mg/L for 40 weeks after the vaccinations. The detailed schedules were shown in **Figures 1A** and **3A**.

### Immunization with DNA and protein vaccines

The preparation of DNA vaccines and liposome was described and validated in our previous papers (30). For protein vaccines, the purification of CTLA-4-PD-L1 was based on our previous experiences (33). In brief, BL21 (DE3) *E. coli* strain transformed with plasmids encoding CTLA-4-PD-L1 was cultured with terrific broth (TB)/ampicillin (100 μg/mL) and grew at 37 °C in a shaker at 250 rpm. When reaching logarithmic phase with OD_600_ at 0.7, the *E. coli* culture was treated 0.1 mM of IPTG and the culture was further incubated at 37 °C overnight. The IPTG induced culture was centrifuged at 6000 rpm for 15 min, and supernatant was removed and the pellet was re-suspended in PBS. The pellet was subjected to continuous high-pressure cell disrupter twice at pressure of 28 kpsi followed by centrifugation at 4500 rpm for 15 min for removal of supernatant. The pellets were washed six-time with either 200 mL of H_2_O or Tris-HCl buffer in the presence or absence of 1%SDC, and 0.5 M NaCl. Each washing steps were followed by centrifugation at 6000 rpm for 15 min. The inclusion body pellets were solubilized with by 8 M urea buffer containing 10 mM imidazole and loaded onto Ni-NTA resin column. Elution of immobilized protein was triggered by imidazole with increasing concentrations of from 10 to 500 mM in PBS buffer (pH 7.5). Fractions containing CTLA-4-PD-L1 were pooled and dilution refolded to a concentration of 0.1 mg/mL by slow dripping into refolding buffer containing NaCl (250 mM), GSH (1 mM) and GSSG (0.1 mM) in Tris-HCl buffer (50 mM, pH 8.5). Regarding the injection, a mixture of 300 μg of DNA vaccines diluted with 5% dextrose in water (w/v) to a final volume of 300 μl was gently mixed with 300μl of liposome and incubated for 25 min at room temperature. For protein vaccines, a mixture of 200 μg of protein accines diluted with PBS to a final volume of 300 μl was gently mixed with 300μl of incomplete Freund’s Adjuvant (IFA, #77145, Thermo Fisher Scientific, Inc.,Waltham, MA, USA). 600 μg DNA vaccine mixture, protein vaccine mixture, or IFA was injected into the muscle at multiple sites (four limbs) once a week for three consecutive weeks.

### Evaluation of treatment efficacy in rats using an animal positron emission tomography

To evaluate the changes in glycolysis in live animals with liver tumors, we conducted 2-deoxy-2-(F-18) fluoro-d-glucose (FDG)-positron emission tomography (PET) studies in rats at the Molecular Imaging Center of Chang Gung Memorial Hospital. In brief, FDG-PET is a reliable detection tool displaying the high glucose metabolism of tumors after fasting for 8 hours (31). The normal liver and tumor tissue’s mean SUV (SUVmean) was determined, and the tumor-to-liver radioactivity ratio was calculated for comparison. The relative SUV (SUVr) in each rat was determined the values of SUVmean at fifth (2^nd^ PET) and ninth (3^rd^ PET) weeks and then normalized itself SUVmean baseline (1^st^ PET).

### Detection of serum antibodies against CTLA-4

The CTLA-4 (PD-L1) 0.5ug/ml in PBS was coated to a 96 well 442404 NUNC-IMMUNO plate (Thermo) 50ul/well at 4°C overnight. After 1 hour of blocking (1%BSA in PBS), the rat serum 1:25 diluted with reagent diluent (0.1%BSA 0.05%Tween-20 in 20mM Tris-base, 150mM NaCl pH7.2-7.4) was added to plate 100ul/well and incubated for 2 hours at room temperature. The goat anti-rat IgG HRP with reagent diluent (1:5000) 100ul/well was applied as a secondary antibody for 1 hour at room temperature. TMB substrate (50ul/well) was applied for 20min and then stopped with 1M H2SO4 (50 μl/well). The absorbance at 450nm was read with a TECAN infinite M200PRO plate reader. Moreover, the plate was washed three times with 0.05%Tween-20 in PBS between each step.

### RNA extraction and quantitative RT-PCR

Tumor samples were homogenized using MagNA Lyser Instrument and MagNA Lyser Green Beads (Roche 03358941001). RNA was extracted using Trizol and then was used for reverse-transcription with HiScript I ^TM^ First Strand cDNA Synthesis Kit (Bionovas, Taipei, Taiwan) according to the manufacturer’s instructions and q-PCR was performed using 1 μl of cDNA. The details of q-PCR were described in our previous study (34). The primers for q-PCR are listed below: GAPDH-F (5’-TCAAATGGGGTGATGCTGGT-3’); GAPDH-R (5’- TCATGAGCCCTTCCACGATG-3’); CD4-F (5’-TTGACCTGTGAGGTGATGGG-3’); CD4-R (5’- GAGCACTGGCAAGTCTTCTTCTC-3’); CD8-F (5’-TTATCACCAAGCCGGTGACG-3’); CD8-R (5’-TGGGACATTTGCAAACACGC-3’); CD274-F (5’-GCCTTCTTGCCAAAGGACCA-3’); CD274-R (5’-GTTGTTTCCCCACTCAGGGA-3’); GZMA-F (5’-ATGTCATGTAGCAGGGTGGG-3’); GZMA-R (5’-AAGCTGTGATGCCTCGGAAA-3’); GZMB-F (5’-GGATGAGTATTCTGGGAGTAAGAAG-3’); GZMB-R (5’-CCAGCCACATAGCACACATCT-3’).

### Statistics

The data are presented as the mean ± SEM. Differences between experimental and control animals were calculated using the Mann–Whitney U test or the Student’s *t*-tests. A constant level of P = 0.05 was used to reject the null hypothesis.

## Results

### The immune response to CTLA4-PD-L1 DNA and protein

In our previous publication, the treatment of CTLA4-PD-L1-I DNA suppressed the tumorigenesis of TAA-induced iCCA in SD rats (30). Peptide/protein-based cancer vaccines do not require the additional steps of transcription and translation for the presentation on dendritic cells (35). Therefore, we constructed a CTLA4-PD-L1-I chimeric protein (Protein vaccine; **Figure S1A**) and evaluated its effect on TAA-induced iCCA tumor growth. The experimental scheme was shown in **Figure 1A**. The baseline tumor sizes were measured using an animal positron emission tomography (PET) system (31). According to the baseline tumor sizes, the iCCA-bearing rats were randomized into three groups. Each group received three weekly intramuscular injections of CTLA-4-PD-L1-I DNA vaccine plus Protein vaccine (two doses of DNA followed by one dose of protein), Protein vaccine alone, or control adjuvant, respectively. The tumor growth was monitored with additional PET scans in weeks 39 and 40. Serum samples were collected before treatment started (1^st^ serum), in week 40 (2^nd^ serum), and in week 44 (3^rd^ serum) and tested for antibody titers. The average rat body weight was similar among the groups, suggesting that CTLA4-PD-L1-I DNA or Protein vaccine treatment did not cause apparent toxicities (**Figure 1B**). Both CTLA-4 antibody titers and PD-L1 antibody titers were increased three weeks after the last dose of the treatment with the combination of CTL-4-PDL1-I DNA and Protein vaccines or Protein vaccine alone (2^nd^ serum). The antibody titers remained steadily increased for additional four weeks (3^rd^ serum) (**Figures 1C, D**), indicating that CTLA-4-PD-L1-I DNA and chimeric protein induced the corresponding endogenous antibodies in TAA-induced iCCA rats.

### Suppression of iCCA tumor growth after the CTLA4-PD-L1-I DNA and chimeric protein treatment

**Figure 2:**
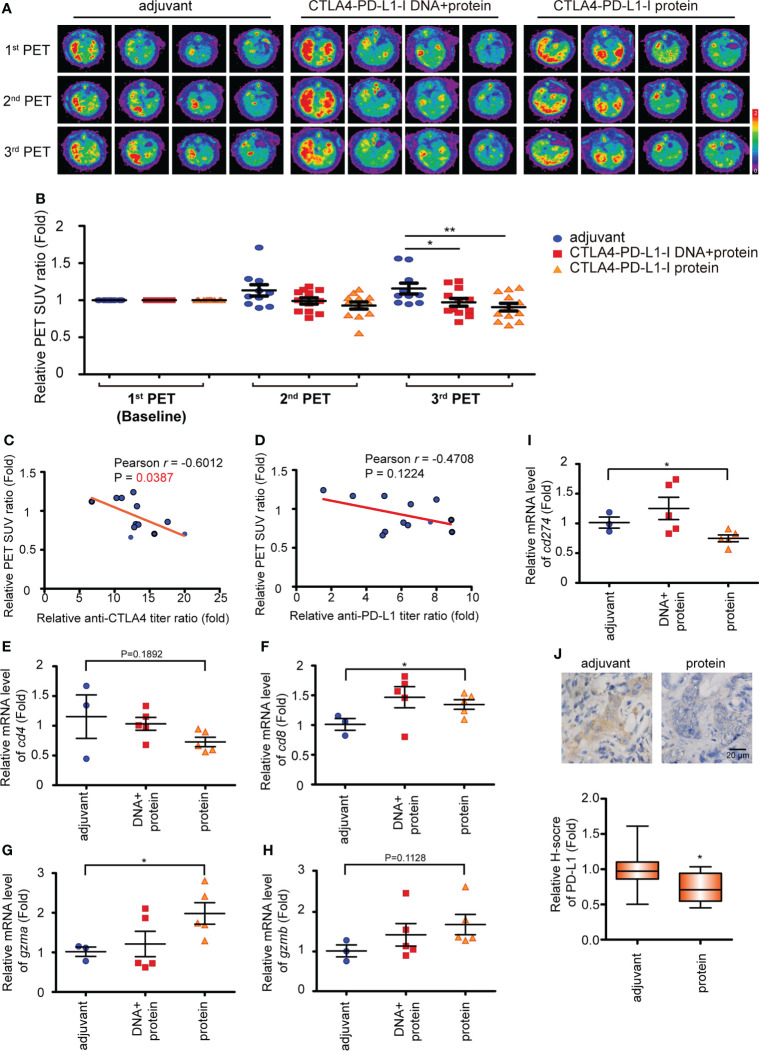
The CTLA4-PD-L1-I DNA or protein vaccine repressed rat iCCA tumor growth. **(A)** Representative image of 18F-FDG microPET on TAA-induced CCA in SD rats. The images were performed before treatment (1^st^ PET) and after five weeks (2^nd^ PET) and nine weeks (3^rd^ PET) of the vaccinations. **(B) **The change of the tumor-to-liver ratio of the standardized uptake value (SUV) in the control adjuvant and experimental groups at five weeks (2^nd^ PET) and nine weeks (3^rd^ PET) after the vaccinations. The values are from two independent experiments and presented as the mean ± SEM. N≧10. *P< 0.05, **P< 0.01 by Student’s *t*-tests. **(C, D)** The correlation between the anti-CTLA4 **(C)** or PD-L1 **(D)** antibody titer ratio and the relative PET SUV ratio. The relative PET SUV ratio from the rats receiving CTLA4-PD-L1-I protein vaccination at nine weeks (3^rd^ PET). The correlation Pearson coefficient *r* and P values are shown in the panel. N=12. **(E-I)** The mRNA expression levels of *cd4* **(E)**, *cd8* **(F)**, *gzma* **(G)**, *gzmb* **(H)**, and *cd274* **(I)** in the tumors from the iCCA-bearing rats receiving a combination of CTLA4-PD-L1-I DNA and protein (DNA + protein), CTLA4-PD-L1-I protein (protein), or control adjuvant. The values (means ± SEM) are presented as the fold-change relative to the average level of the tumors from the rats receiving control adjuvant. N≧3.*P < 0.05 by Student’s *t*-test. **(J)** Upper: Immunohistochemical staining of PD-L1 in TAA-induced iCCA receiving CTLA4-PD-L1-I protein (protein) or adjuvant. Scale bars: 20 μm. Lower: The values (means ± SEM) are presented as the fold-change relative to the average H score of the tumors from the rats receiving control adjuvant. N≧10.*P < 0.05 by Student’s *t*-test.

In order to track tumor growth, the SUV intensities of iCCA were determined on PET scans in week 39 (2^nd^ PET) and week 43 (3^rd^ PET). The SUV intensities were reduced in the tumor-bearing rats receiving the combination of CTLA-4-PD-L1-I DNA and Protein vaccines or Protein vaccine alone in week 43 (**Figures 2A, B**), suggesting suppression of tumor growth, which correlated with the increased CTLA-4 and PD-L1 antibody titers as shown in **Figures 1C** and **D**. A negative correlation between anti-CTLA4 titer ratio and the relative PET SUV ratio was noted in the rats receiving Protein vaccine alone (p=0.0387; **Figure 2C**). However, only a trend of negative correlation was found between the changes of anti-PD-L1 titer ratio and the relative PET SUV ratio (p=0.1224; **Figure 2D**). Treatment with Protein vaccine alone increased tumor infiltration of CD8^+^ T cells (**Figure 2F**) but not CD4^+^ T cells (**Figure 2E**; p=0.1892). The mRNA expression level of granzyme A (*gzma*) was also upregulated in rats receiving CTLA4-PD-L1 protein (**Figure 2G**), while granzyme B (*gzmb*) only show a trend of increase (**Figure 2H**). In contrast, PD-L1 (*cd274*) expression was reduced after the treatment of Protein vaccine in terms of mRNA (**Figure 2I**) and protein (**Figure 2J**) levels.

### CTLA4-PD-L1-II chimeric protein induced a more sustained antibody titers

**Figure 3:**
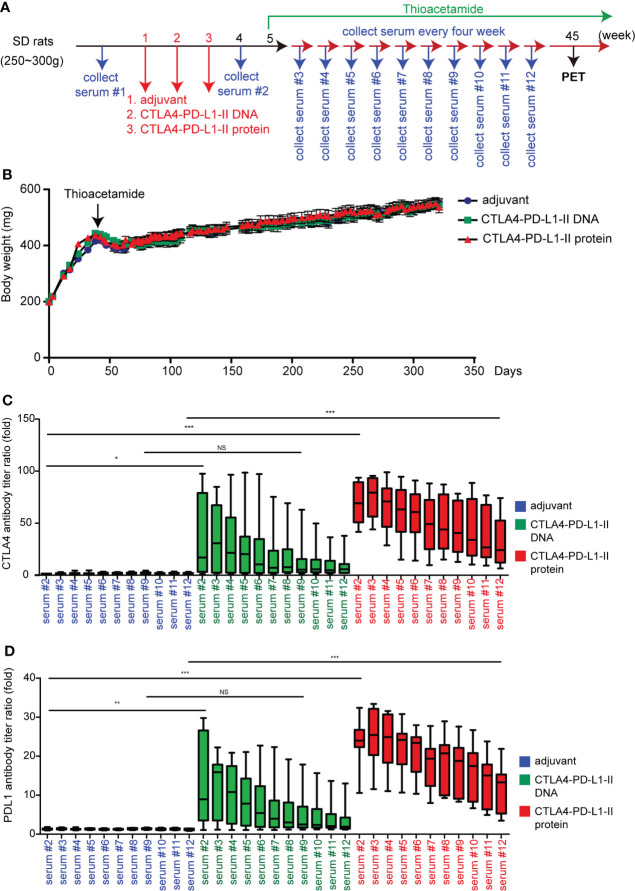
The effect of CTLA4-PD-L1-II DNA or protein vaccine. **(A)** A schema for experimental design for **Figure 3**. **(B)** The average* *body weight of rats (N=9) receiving CTLA4-PD-L1-II DNA, protein vaccines, or control adjuvant (Freund’s adjuvant). **(C, D) **The antibody titers of CTLA4 **(C)** or PD-L1 **(D)** were determined after one week (serum #2) of CTLA4-PD-L1-II DNA (DNA), CTLA4-PD-L1-II protein (protein, or control adjuvant, and the antibody titers detected every four weeks for another 40 weeks (serum #3 – serum #12). N=9. Box-and-whisker plots show the data distribution: maximum, upper quartile, median, lower quartile, and sample minimum. *P< 0.05, **P< 0.01, ***P< 0.001 by Student’s *t*-tests. NS, not significant.

In order to validate whether CTLA4-PD-L1 DNA or Protein vaccine acts as a tumor vaccine for iCCA, these reagents were injected before iCCA induction by TAA, i.e., in a preventive setting. Because the increased anti-PD-L1 antibody level was not significantly correlated with the decreased PET SUV ratio (**Figure 2D**), a more extended coding sequence of PD-L1 was cloned in the DNA construct, resulting in CTLA4-PD-L1-II DNA and CTLA4-PD-L1-II protein (Protein vaccine-II), in an attempt to generate more potent anti-PD-L1 antibodies (**Figure S1B**). The first rat serum (serum #1) was collected as the baseline antibody titers before the injection. The baseline tumor sizes were measured by an animal PET system. CTLA-4-PD-L1-II DNA, Protein vaccine-II, and control adjuvant were injected once a week for three consecutive weeks. The second serum (serum #2) was collected in week 4 to evaluate the immune response to the vaccines, and then the rats were fed with drinking water containing TAA to induce iCCA. During iCCA development, serum samples were collected every four weeks for up to 40 weeks (serum #3 - serum #12). The PET was performed in week 45 to evaluate the tumor response (**Figure 3A**). The average body weight was still not affected by the treatment with CTLA-4-PD-L1-II DNA vaccine or Protein vaccine-II (**Figure 3B**). After CTLA-4-PD-L1-II DNA or Protein vaccine-II treatment, the following serum samples showed increased anti-CTLA-4 and anti-PD-L1 antibody titers, which gradually decreased with time (**Figures 3C, D**). Notably, the antibody titers induced by CTLA4-PD-L1-II DNA treatment decreased to baseline level by 30 weeks after treatment (as shown in serum #7), while those induced by Protein vaccine-II remained increased till the end of experiment (as shown in serum #12) (**Figures 3C, D**).

### CTLA4-PD-L1-II chimeric protein inhibits rat iCCA tumorigenesis induced by TAA

**Figure 4:**
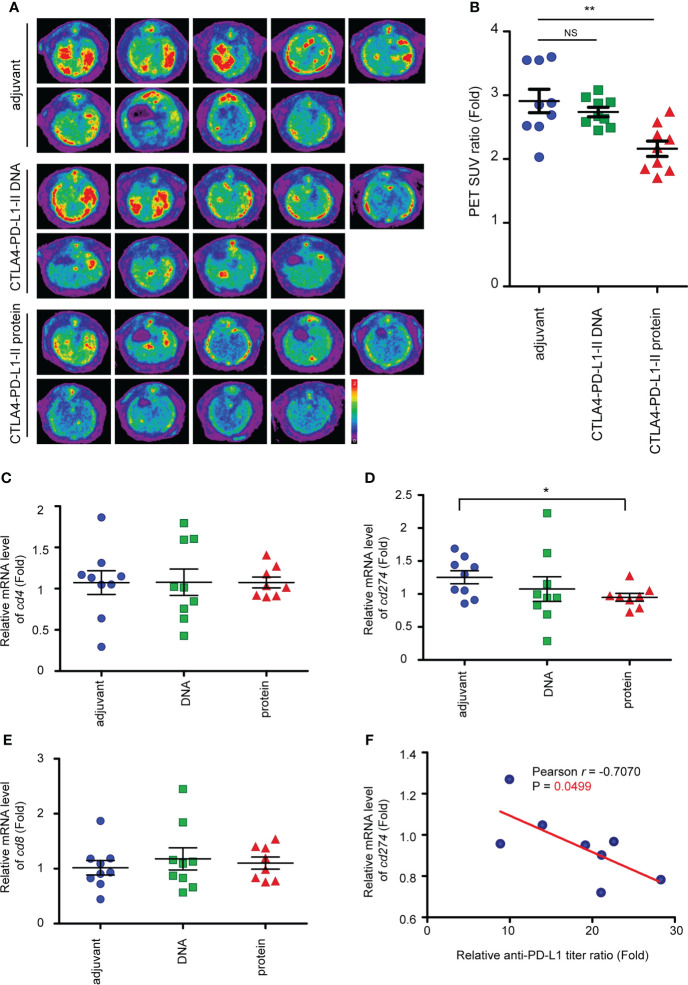
Reduced of iCCA tumorigenesis after CTLA4-PD-L1-II chimeric protein vaccination. **(A)** Representative image of 18F-FDG microPET on TAA-induced CCA in SD rats. The images were performed at week 45 after the treatment of CTLA4-PD-L1-II DNA, chimeric protein, or control adjuvant. **(B)** The tumor-to-liver ratio of SUV in the control adjuvant and experimental groups at 45 after the vaccination. The values are presented as the mean ± SEM. N=9. **P< 0.01 by Mann–Whitney U tests. NS, not significant. **(C–E)** The mRNA expression levels of *cd4* **(C)**, *cd8* **(D)**, and *cd274* **(E)** in the tumors from the rats receiving the vaccination of CTLA4-PD-L1-I DNA or CTLA4-PD-L1-I protein, or control adjuvant. The values (means ± SEM) are presented as the fold-change relative to the average level of the tumors from the rats receiving control adjuvant. N=9.*P < 0.05 by Student’s *t*-test. **(F)** The correlation between the anti-PD-L1 antibody titer ratio and the relative mRNA level of *CD274*. The correlation Pearson coefficient *r* and P values are shown in the panel. N=9.

To track tumor development, the SUV intensities of iCCA were determined in week 45 (the schema shown in **Figure 3A**). Interestingly, the SUV intensities decreased in the rats receiving Protein vaccine-II, but not CTLA4-PD-L1-II DNA (**Figures 4A, B**), suggesting Protein vaccine-II may serve as a tumor vaccine to prevent TAA-induced tumorigenesis of iCCA in rats. Notably, the amount of CD8^+^ T cells and CD4^+^ T cells in the iCCA tumors were not affected by the treatment with CTLA4-PD-L1-II DNA or Protein vaccine-II (**Figures 4C, D**).

PD-L1 (*cd274*) mRNA expression level was reduced after the treatment with Protein vaccine-II (**Figure 4E**). Furthermore, a negative correlation between the mRNA levels of *cd274* and the relative anti-PD-L1 antibody titer ratio was found in the rats receiving Protein vaccine-II (**Figure 4F**).

**Figure 5:**
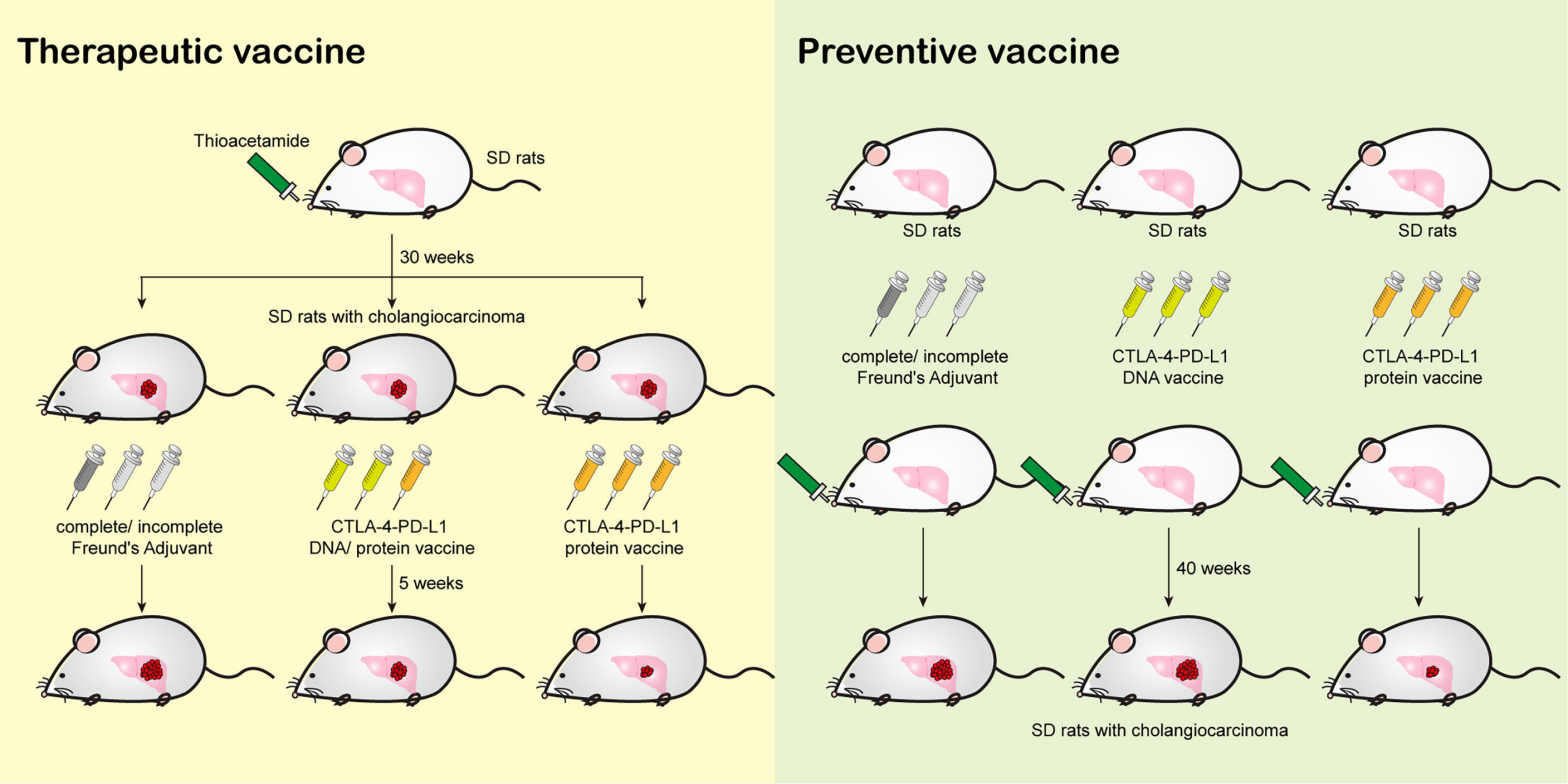
A diagram summarizing the major findings in this study.

Here, we summarize the findings shown in **Figure 5**, we demonstrated the effect of the vaccinations on tumorigenesis in a TAA-induced iCCA rat model.

## Discussion

Several clinical studies using antibodies against immune checkpoints, such as CTLA-4, PD-1, and PD-L1, had shown excellent results in numerous types of cancers (36–41). We previously reported that vaccination with a CTLA4-PD-L1 DNA cancer vaccine induced endogenous antibodies against CTLA-4 and PD-L1, and inhibited the tumor growth in a spontaneous TAA-induced rat iCCA model (29). In this study, we constructed a CTLA4-PD-L1 chimeric protein vaccine (Protein vaccine), and compared its effects with the DNA vaccine. Vaccination with Protein vaccine alone induced significant increase of CTLA4 and PD-L1 antibody titers, and interestingly, the extent of increase was as effective as that achieve by combined DNA plus Protein vaccinations (**Figures 1C, D**).

In a therapeutic setting, iCCA-bearing rats received either DNA plus Protein vaccines or Protein vaccine alone (the scheme shown in **Figure 1A**), and the changes in tumor burden were measured with PET scans (**Figure 2A**). Similar to the trend in antibody induction, treating iCCA-bearing rats with Protein vaccine alone resulted in a tumor inhibition (shown as a decreased SUV ratio) at least as potent as that achieved by combined DNA plus Protein vaccinations (**Figure 2B**). In addition, Protein vaccination alone led to the increase of CTAL4 antibody titers that correlated with the decrease of tumor burden (SUV ratio), along with increased *cd8* and granzyme A (*gzma*) expression, and decreased PD-L1 expression on tumor cells (**Figures 2C–J**).

Next, to test whether our immune checkpoint DNA or Protein vaccine has a cancer preventive function, DNA or Protein vaccines were injected in rats before the induction of iCCA by TAA (**Figure 3A**). Compared with DNA vaccines, Protein vaccines induced a more sustained PD-L1 and CTLA-4 antibody titers, lasting up to 40 weeks after the vaccinations were completed (**Figures 3C, D**). Protein vaccine treatment inhibited the tumorigenesis of iCCA in rats despite being fed continuously with TAA (**Figures 4A, B**). Correspondingly, Protein vaccines led to the downregulation of PD-L1 gene expression in the tumor samples, the extent of which correlated with the increase of anti-PD-L1 titers (**Figures 4E, F**). It is an intriguing future topic to clarify the mechanisms by which Protein vaccine modulate the PD-L1 expression in the tumor microenvironment.

The combined therapy has been investigated to improve response rates in biliary tract cancers (BTCs) (42). Several preclinical studies and clinical trials have been shown that immune checkpoint inhibitors should be combined with chemotherapies, radiotherapies, and targeted therapies in BTCs (43–45). Those combination therapies have been reported to increase the response rates in BTCs. TOPAZ-1 demonstrated that durvalumab (anti-PD-L1) plus chemotherapy improved overall survival in patients with BTCs (19). This current study regarded CTLA4-PD-L1 DNA and protein vaccines as therapeutic cancer vaccines. A combination of CTLA4-PD-L1 cancer vaccines and chemotherapies, radiotherapies, or targeted therapies may enhance therapeutic effects in BTCs, which needs to be done in future studies

The cost of immunotherapy has been expensive. Our DNA and proteins vaccines aim to continuously induce the antibodies of CTLA4 and PD-L1. The cancer vaccines may decrease patients’ financial burden for immunotherapies. Additionally, the indications for inhibitors of immune checkpoint proteins are comprehensive, potentially including all major cancer types, such as prostate, lung, pancreatic, liver cancers, and so on (4, 46).

In conclusion, we have developed a CTLA4-PD-L1 chimeric protein vaccine, which may function both as a therapeutic cancer vaccine and as a preventive cancer vaccine in the TAA-induced iCCA rat model.

## Data availability statement

The raw data supporting the conclusions of this article will be made available by the authors, without undue reservation.

## Ethics statement

The animal study was reviewed and approved by Institutional Animal Care and Use Committee of Chang Gung Memorial Hospital.

## Author contributions

Y-RP: Validation, Formal analysis, Investigation, Writing - Original Draft; C-EW: Conceptualization, Investigation, Funding acquisition, Writing - Review & Editing; W-KH: Conceptualization, Investigation, Data Curation, Writing - Review & Editing; M-HC: Investigation, Supervision; K-HL: Validation, Writing - Review & Editing, Visualization, Supervision; C-NY: Conceptualization, Methodology, Supervision, Writing - Review & Editing, Funding acquisition. All authors contributed to the article and approved the submitted version.

## Funding

This work was supported by grants from Linkou Chang-Gung Memorial Hospital (NMRPG3F6021~2, CMRPG3K0711, CMRPG3I0231, and CRRPG3K0011~2 to CNY and CRRPG3K0021~2 to CEW), National Taiwan University Hospital (111-S0227 to KHL) and the Ministry of Science and Technology (105-2314-B-182A-041-MY2 to CNY).

## Acknowledgments

Thanks for technical assistance from Laboratory Animal Center and Center for Advanced Molecular Imaging and Translation and Common Laboratory, Chang Gung Memorial Hospital, Linkou.

## Conflict of interest

The authors declare that the research was conducted in the absence of any commercial or financial relationships that could be construed as a potential conflict of interest.

## Publisher’s note

All claims expressed in this article are solely those of the authors and do not necessarily represent those of their affiliated organizations, or those of the publisher, the editors and the reviewers. Any product that may be evaluated in this article, or claim that may be made by its manufacturer, is not guaranteed or endorsed by the publisher.
