# Supplementary material for: Chimeric immune checkpoint protein vaccines inhibit the tumorigenesis and growth of rat cholangiocarcinoma
Source: Front Immunol. 2022 Oct 20;13:982196. doi: 10.3389/fimmu.2022.982196 (PMC9631822; doi:10.3389/fimmu.2022.982196)
Supplement: Supplementary file 1 [file DataSheet_1.docx]

**Supplementary materials**


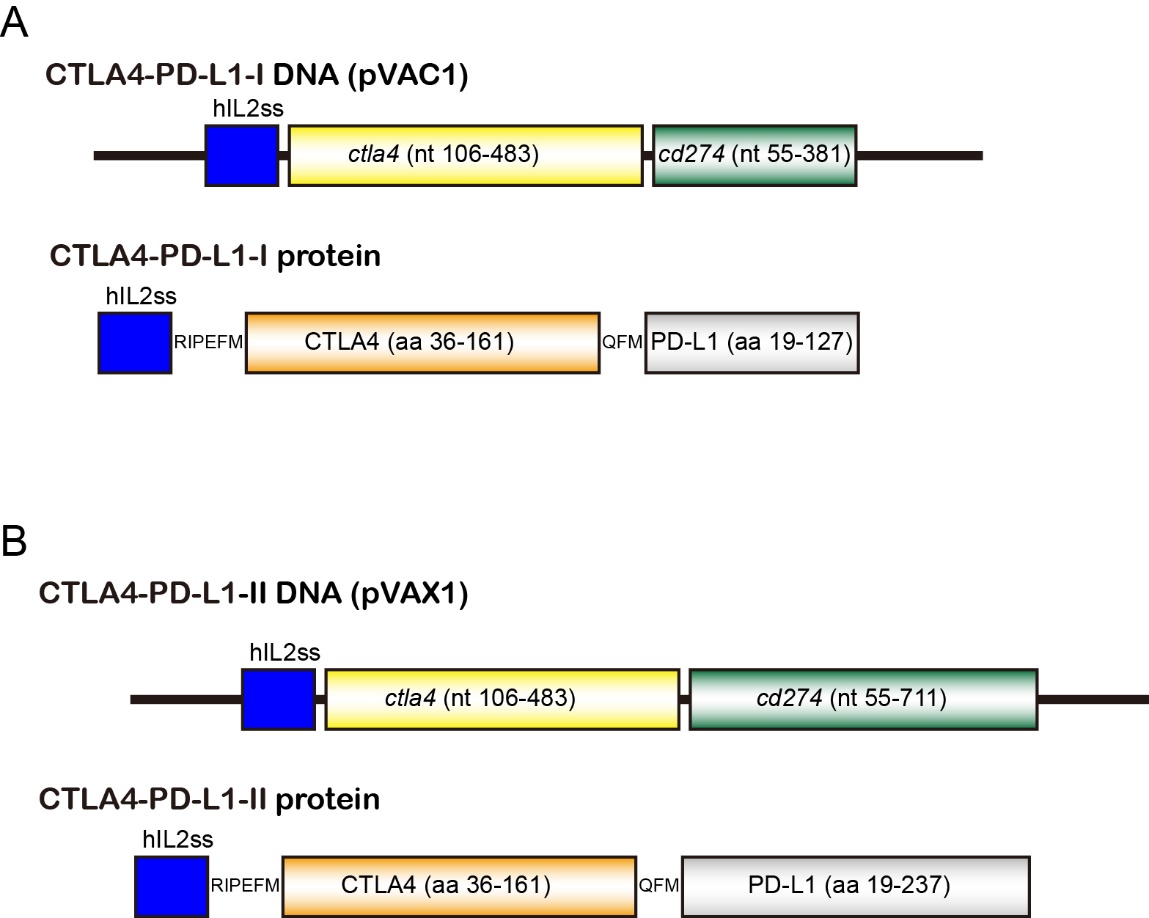


**Supplementary Figure 1. Schemas of the constructs**

1. Schemas for the design of CTLA4-PD-L1-Ⅰ DNA vaccine and CTLA4-PD-L1-Ⅰ protein vaccine
2. Schemas for the design of CTLA4-PD-L1-Ⅱ DNA vaccine and CTLA4-PD-L1-Ⅱ protein vaccine
